# SNCA is a potential therapeutic target for COVID-19 infection in diffuse large B-cell lymphoma patients

**DOI:** 10.1007/s10495-024-01996-9

**Published:** 2024-07-15

**Authors:** Can Chen, Yun Li, Yiwei Li, Zhenzhen Chen, Pengfei Shi, Yaping Xie, Shenxian Qian

**Affiliations:** 1 Department of Hematology, Affiliated Hangzhou First People’s Hospital, School of Medicine, Westlake University, Hangzhou, China; 2 Team of Neonatal & Infant Development, Health and Nutrition, NDHN. School of Biology and Pharmaceutical Engineering, Wuhan Polytechnic University, Wuhan, China; 3 Kindstar Global Precision Medicine Institute, Wuhan, China

**Keywords:** DLBCL, SNCA, COVID-19, Dendritic cells, Biomarker

## Abstract

Cuprotosis related genes (CRGs) have been proved to be potential therapeutic targets for coronavirus disease 2019 (COVID-19) and cancer, but their immune and molecular mechanisms in COVID-19 infection in Diffuse Large B-cell Lymphoma (DLBC/DLBCL) patients are rarely reported. Our research goal is first to screen the key CRGs in COVID-19 through univariate analysis, machine learning and clinical samples. Secondly, we determined the expression and prognostic role of key CRGs in DLBCL through pan-cancer analysis. We validated the expression levels and prognosis using multiple datasets and independent clinical samples and validated the functional role of key CRGs in DLBCL through cell experiments. Finally, we validated the expression levels of CRGs in COVID-19 infected DLBCL patients samples and analyzed their common pathways in COVID-19 and DLBCL. The results show that synuclein-alpha (SNCA) is the common key differential gene of COVID-19 and DLBCL. DLBCL cells confirm that high expression of SNCA can significantly promote cell apoptosis and significantly inhibit the cycle progression of DLBCL. High expression of SNCA can regulate the binding of major histocompatibility complexes (MHCs) and T cell receptor (TCR) by regulating immune infiltration of Dendritic cells, effectively enhancing T cell-mediated anti-tumor immunity and clearing cancer cells. In conclusion, SNCA may be a potential therapeutic target for COVID-19 infection in DLBCL patients. Our study provides a theoretical basis for improving the clinical treatment of COVID-19 infection in DLBCL patients.

**Supplementary Information:**

The online version contains supplementary material available at 10.1007/s10495-024-01996-9.

## Introduce

Coronavirus disease 2019 (COVID-19) is an acute respiratory infectious disease caused by SARS CoV-2, SARS-CoV-2 is a single-stranded RNA β-coronavirus, four types of proteins and lipid membranes are involved in its pathogenicity [1]. According to the data of the World Health Organization (WHO), since December 2019, COVID-19 infection has spread all over the world [2]. At present, there are more than 760 million cases and 6.9 million deaths, but the actual number is considered higher, the virus mutation has not stopped yet [3]. Based on the current epidemiological investigation and research results, the main infectious sources of COVID-19 are asymptomatic and symptomatic infected persons [4]. Asymptomatic infection means that some patients usually have no obvious clinical symptoms after infection, but the detection result of novel coronavirus nucleic acid test is positive. Symptomatic carriers refer to some patients who may experience symptoms such as fever, cough, fatigue, and difficulty breathing after infection. COVID-19 infection is more serious with symptoms than without symptoms [5].

Cancer patients are more susceptible to COVID-19 infection, and those with lung cancer, hematological malignancies and metastatic diseases have the highest incidence of serious events [6], as well as those who receive stem cell transplantation and cell therapy [7]. There are many complex interactions between COVID-19 and cancer [8]. Some studies have found that there is an interaction between inflammatory reaction and immunity in patients with COVID-19 and Lung adenocarcinoma (LUAD) [9]. Another study has found that the spike protein S1 of SARS-CoV-2 can cause functional changes in lung cancer cells [10]. Some susceptibility factors caused by SARS-CoV-2 may promote the development and occurrence of Diffuse Large B-cell Lymphoma (DLBC/DLBCL) [11]. DLBCL is more likely to be infected by COVID-19 due to decreased immune function, and DLBCL has also been reported as a high-risk factor of COVID-19 [12]. COVID-19 and DLBCL share a common signal crosstalk mechanism [13], but the specific mechanism has not yet been elucidated.

In 2022, a research report in Science showed that copper induces cell death (cuprotosis) is a novel mechanism of cell death, it occurs through the direct binding of lipoylation components in the tricarboxylic acid (TCA) cycle with copper. This results in lipoylated protein aggregation and subsequent iron-sulfur cluster protein loss leading to proteotoxic stress and ultimately cell death [14]. More and more research evidence shows that the serum copper ion level of patients with COVID-19 is increased [15]. Compared with non-severe patients, the whole blood copper level of severe patients with COVID-19 is significantly increased [16]. The imbalance of copper homeostasis in the body is closely related to the occurrence and development of cancer, copper ion levels in samples of different types of tumor tissues showed an upward trend, including DLBCL, breast cancer, lung cancer and digestive tract renal cancer [17]. The level of copper ions in patients with COVID-19 and cancer is elevated, and excessive copper ions can induce tumor cell death [18], therefore, cuprotosis plays a complex role in the development of COVID-19 and cancer. Cuprotosis related genes (CRGs) have been shown to be potential therapeutic targets for COVID-19 and cancer [19, 20]. The cuprotosis related DLD gene has been proven to be a potential therapeutic target for COVID-19 infected DLBCL patients [21], suggesting that CRGs play an important role in the mechanism of COVID-19 infected DLBCL patients. Therefore, a deeper understanding of cuprotosis and its related biological processes is of great significance for developing new treatment strategies for COVID-19 infected DLBCL patients.

## Materials and methods

### Microarray data acquisition and processing

The gene expression dataset for synuclein-alpha (SNCA) expression level evaluation includes GSE177477, GSE176405, GSE56315, GSE25638, TCGA-DLBC. The dataset used to evaluate SNCA as a potential prognostic marker after DLBCL treatment includes TCGA-DLBC and GSE181063. The Cancer Genome Atlas (TCGA) database (https://www.cancer.gov/ccg/research/genome-sequencing/tcga) and The GEO dataset can be downloaded from the Gene Expression Comprehensive Database (GEO) (https://www.ncbi.nlm.nih.gov/geo/). The GSE177477 was obtained from GPL23195 platform, including 29 SARS-CoV-2 positive case samples (11 symptomatic patients and 18 asymptomatic patients) and 18 healthy control samples in respiratory tract samples of COVID-19 patients, The GSE176405 was obtained from GPL16699 platform, including 6 samples of bronchial epithelial cells from SARS-CoV-2 positive patients and 7 samples of bronchial epithelial cells from healthy individuals. The GSE56315 dataset was obtained from the GPL570 platform, including 33 matched normal samples and 55 DLBCL samples. The GSE25638 dataset was obtained from the GPL570 platform, including 13 matched normal samples and 26 DLBCL samples. TCGA-DLBC dataset includes 47 DLBCL samples, Genotype-Tissue Expression (GTEx) dataset (https://www.gtexportal.org) includes 337 normal samples. The GSE181063 dataset was obtained from the GPL14951 platform, including 1311 confirmed DLBCL formalin fixed paraffin embedded (FFPE) samples. For datasets from GEO databases, the raw data were downloaded as MINiML files, it contains the data for all platforms, samples and GSE records of the GSE. The extracted data were normalized by log2 transformation. The microarray data were normalized by the normalize quantiles function of the preprocessCore package in R software (version 4.2.1). Probes were converted to gene symbols according to the annotation information of the normalized data in the platform. Probes matching multiple genes were removed out from these datasets, The average expression value of gene measured by multiple probes was calculated as the final expression value. As in the case of the same dataset and platform but in different batches, used the removeBatchEffect function of the limma package in the R software to remove batch effects. After there is no batch effect in the data, subsequent differential analysis will be conducted. We used the R 4.2.1 tool to evaluate prognostic risk assessment and gene expression levels [21]. 62 CRGs were obtained from previously published literature [22, 23].

### GEO database and machine learning used to screen key CRGs of COVID-19

we obtained data from GEO177477 database and performed gene expression analysis. After data standardization, If the data has no batch effect, it can be used as a batch of data for subsequent analysis. Before using machine learning (Random Forest, RF) model to screen for key CRGs, we used the limma package in R software to conduct preliminary screening of differentially expressed genes. In order to retain as many potentially valuable genes as possible, we used a relatively relaxed threshold for preliminary screening of genes. “P < 0.05 and Fold change (FC) > 1.50 or FC < 0.67” is defined as the screening threshold for differentially expressed genes. A machine that learnt model was subsequently used to further screen and select the gene with the highest contribution to classification as the key CRGs, compared with the conventional modeling method, RF attracted increasing attention due to its accuracy and high precision. We developed an RF model with ntree = 500 and mtry = 3 using the randomForest package in R software. IncNodePurity > 0.6 as the threshold for screening key CRGs [24].

### Independent dataset to validate key genes of COVID-19

We first used COVID-19 samples from the GSE177477 dataset to analyze the differential genes (*P* < 0.05 and FC > 1.50 or < 0.67), A RF model was subsequently used to further screen and select the gene with the highest contribution to classification (IncNodePurity > 0.6) as the key gene, and then used R soft 4.2.1 to analyze the GSE176405 dataset to verify GSE177477 dataset’s results To evaluate the potential correlation between SNCA and the severity of COVID-19 patients, we analyzed the expression level of SNCA based on 18 asymptomatic infection patient samples and 11 symptomatic infection patient samples from GSE177477 [25].

### Differential expression and prognostic value of SNCA in pan-cancers

To confirm that SNCA is a potential oncogene, we analyzed SNCA’s pan-cancer expression level through the TCGA database. Gene Expression Profiling Interaction Analysis (GEPIA, http://gepia.cancer-pku.cn/index/html) can be used to evaluate the SNCA expression data of 8587 normal samples and 9736 tumor samples in the TCGA database and GTEx data. When obtaining SNCA’s gene expression profile, Analysis of Variance method was used to compare with the following thresholds: | log2FC | cutof = 1 and q value cutof = 0.01. The ‘Survival Map’ module from the GEPIA online tool was used to select all TCGA malignancies in which SNCA expression significantly correlated with overall survival (OS). For these cancer types, samples were categorized into a high-expression or a low-expression subgroup for subsequent Kaplan–Meier survival analysis according to SNCA expression [26].

### SNCA is a potential biomarker for DLBCL

DLBCL samples from the TCGA-DLBC dataset to analyze the key regulated genes with significant differences between the two diseases. Then it was confirmed through GSE56315 and GSE25638 (P < 0.05 and FC > 1.50 or FC < 0.67” are considered to have significant differences). In order to evaluate SNCA’s prognostic role in DLBCL, based on the TCGA-DLBC and GSE181063 dataset, the prognosis of DLBCL patients after treatment was evaluated by analyzing SNCA’s high and low expression with Kaplan-Meier curve [27].

### Construction of SNCA overexpression DLBCL cell model

OCI-LY1 cells are sourced from the cell bank of Hangzhou First Hospital in Zhejiang Province. OCI-LY1 cells are cell lines of GCB type DLBCL. OCI-LY1 cells were inoculated in DMEM medium containing 10% fetal bovine serum (containing 100 U/mL penicillin and 100 mg/mL streptomycin) and cultured at 37 ℃ in 5% CO2 incubator. When the adherent parietal cell grows into a compact monolayer, it is subcultured. Partial stably growing DLBCL cancer cells were randomly divided into two groups: the empty control group (OCI-LY1 + NC) and the SNCA overexpression group (OCI-LY1 + SNCA-OE). The SNCA overexpression plasmid vector and the nonsense sequence SNCA plasmid vector were transfected into DLBCL cells, respectively. Besides, qPCR was used to verify SNCA’s overexpression effect in OCI-LY1 cells [28].

### Cell apoptosis experiment

Cells were collected (1 × 10^6^ cells/time) and washed with pre-cooled PBS. Then we resuspended the cells using 1 ml 1X Binding Buffer and achieved a density of 1 × 10^6^ cells/ml in the tube. Then, 5 µ L Annexin V-FITC was added to the tube and gently mix for 10 min at room temperature and in dark conditions. Finally, 5 µ L Propidine iodide (PI) was added to the tube for incubation and at room temperature and in dark conditions for 5 min, and then detected by flow cytometry within 1 h. [29].

### Cell cycle experiment

After corresponding culture stimulation, OCI-LY1 cells and culture medium will be transferred to centrifuge tubes. The centrifuge tubes will be centrifuged at 4 ℃ for 5 min (1000 rpm) and the supernatant will be removed, 3 ml of pre-cooled PBS was added to a centrifuge tube to resuspend cells. The centrifuge tube was centrifuged at 4 ℃ for 5 min (1000 rpm) and the supernatant was removed, pre-cooled 75% alcohol was added to a centrifuge tube to resuspend and fix the cells and placed in a refrigerator at 4 ℃ for overnight fixation. Subsequently, pre-cooled PBS was added to the centrifuge tube and washed three times. The centrifuge tube was centrifuged at 4 ℃ for 5 min (1000 rpm) before removing the supernatant. Finally, PI staining solution was added and stained in the dark at 37 ℃ for 30 min before flow cytometry detection [30].

### Quantitative reverse transcription polymerase chain reaction

SNCA were further identified for validation. A total of 18 subjects were recruited in this study, including 13 patients from Hangzhou First People’s Hospital (5 COVID-19 subjects, 4 DLBCL subjects not infected with COVID-19, and 4 DLBCL subjects infected with COVID-19), and 5 healthy volunteers. All patients gave informed consent before the start of the study. Peripheral blood samples were collected from each participant prior to initial treatment. Total RNA was extracted from the peripheral blood mononuclear cells (PBMC) samples using TRIzol reagent (Vazyme, Cat: R401-01). Reverse transcription was performed using the HiScript III RT SuperMix for qPCR (+ gDNA wiper) (Vazume). Next, qPCR was performed using ChamQ Universal SYBR qPCR Master Mix (Vazume) based on LightCycler^®^ 480 II Real-Time PCR System (Roche). GAPDH as an internal reference. The 2-ΔΔCt method was used to determine the relative SNCA expression, FC and Wilcoxon rank sum test was used for comparison between the two groups [31].

### Pathway analysis of SNCA at the transcriptional level in COVID-19 and DLBCL

To analyze the common pathway of the SNCA in DLBCL and COVID-19, the psych package in R software is used to calculate the Spearman correlation between SNCA and other genes. Spearman correlation analysis uses all patient samples from the dataset, correlation coefficient *R* > 0 represents a positive correlation, while correlation coefficient *R* < 0 represents a negative correlation. We screened genes closely related to SNCA based on the TCGA-DLBC dataset and GSE177477 dataset (correlation coefficient | R |>0.3 and *P* < 0.05), and then used these screened genes for gene set enrichment analysis (GSEA) analysis. R package ClusterProfiler package is used to analyze the GSEA. GSEA enrichment analysis uses all patient samples from the dataset, False discovery rate (FDR) < 0.2 and normalized P value < 0.05 were set as the threshold values. [27, 28].

### Correlation analysis between SNCA and immune cell infiltration in COVID-19 and DLBCL

Based R package (immunedeconv R package), We used TIMER method to determine whether the expression of SNCA in COVID-19 and DLBCL is significantly correlated with the immune cell infiltration (CD4 + T cells, B cells, CD8 + T cells, neutrophils, dendritic cells (DCs) and macrophages) in DLBCL and COVID-19, *p* < 0.1 was considered statistically to be significant [32].

## Result

### GEO database and machine learning used to screen key CRGs of COVID-19

**Fig. 1:**
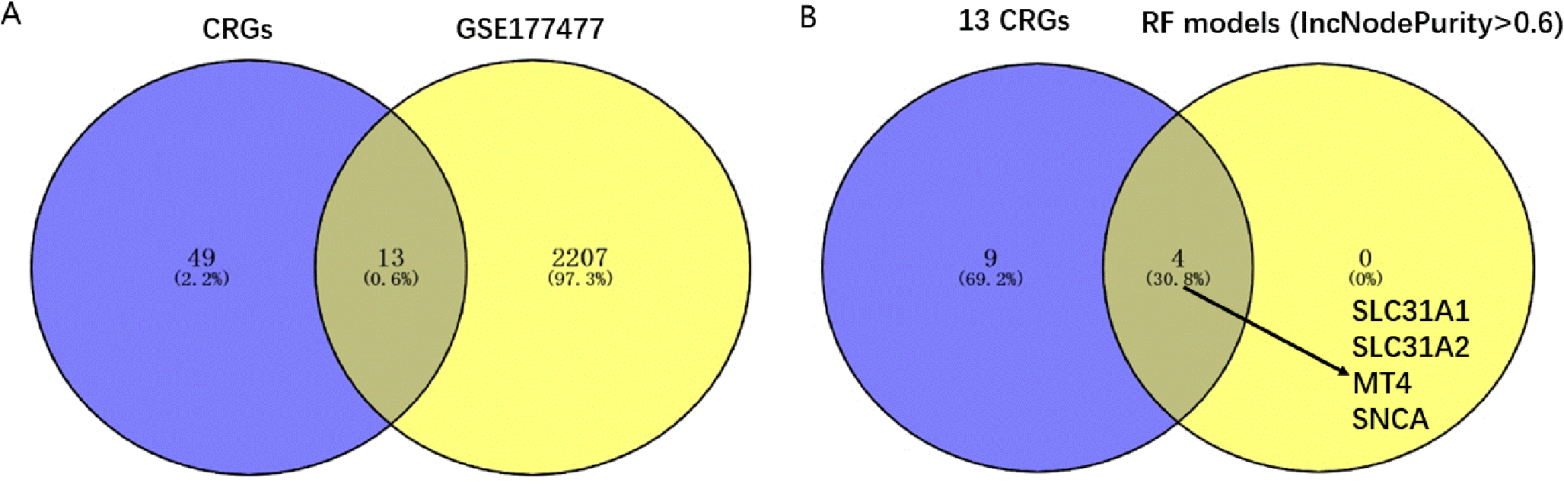
GSE177477 and machine learning used to screen key CRGs of COVID-19. The results were shown 13 Cuprotosis related genes (CRGs) were considered to have significant differences (Fold change (FC) > 1.5 or FC < 0.67 and *P* < 0.05) (**A**). Random Forest models were used to further screen the genes with the highest contribution to classification (IncNodePurity > 0.6) as key genes. SLC31A1, SLC31A2, MT4 and SNCA were considered to be the genes with the highest contribution to classification (**B**)

We obtained data from GEO177477 and conducted gene expression analysis. After data standardization, 13 CRGs were considered to have significant differences (*P* < 0.05 and FC > 1.5 or FC < 0.67) (Fig. 1A). Subsequently, RF models were used to further screen the genes with the highest contribution to classification (IncNodePurity > 0.6) as key genes. SLC31A1, SLC31A2, MT4 and SNCA were considered to be the genes with the highest contribution to classification (Fig. 1B).

### Independent dataset and clinical samples to validate key genes of COVID-19

**Fig. 2:**
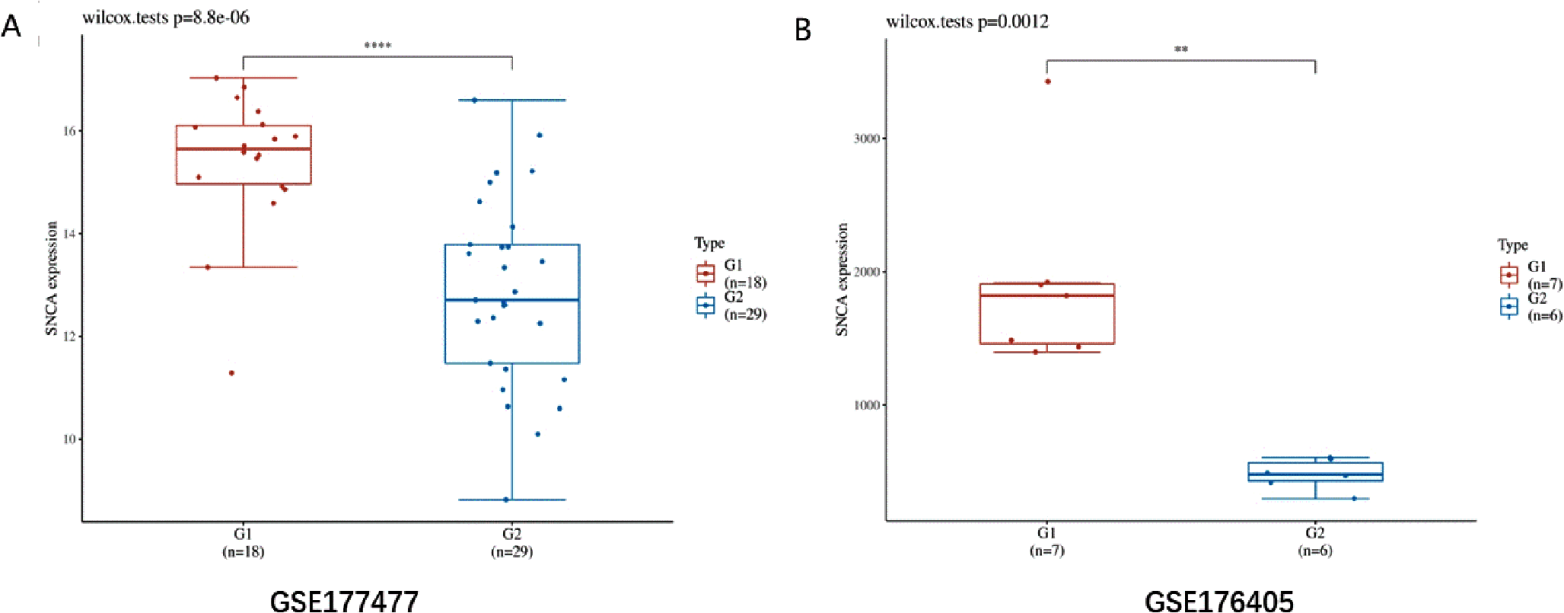
Independent dataset to validate key genes of COVID-19. SNCA was significantly downregulated in GSE177477 datasets (**A**). SNCA was significantly downregulated in GSE176405 datasets (**B**) (G1 group and red represent healthy samples, G2 group and blue represent COVID-19 samples, *P* < 0.05 is considered to have significant differences)

We first used COVID-19 samples from the GSE177477 dataset to analyze the differential genes (*P* < 0.05 and FC > 1.50 or < 0.67), A RF model was subsequently used to further screen and select the gene with the highest contribution to classification (IncNodePurity > 0.6) as the key gene, and then used R soft 4.2.1 to analyze the GSE176405 dataset to verify GSE177477 dataset’s results. SNCA was significantly downregulated in two independent datasets (Fig. 2A-B), SLC31A1, SLC31A2 and MT4 are not significantly differentially expressed in the GSE176405 dataset (Figure S1) (G1 group and red represent healthy samples, G2 group and blue represent COVID-19 samples, *P* < 0.05 and FC > 1.50 or < 0.67 is considered to have significant differences). The qPCR detection results of clinical samples showed that compared with the control group, the expression level of SNCA in patients with COVID-19 was significantly down regulated (Figure S2).

### Differential expression and prognostic value of SNCA in pan-cancers

**Fig. 3:**
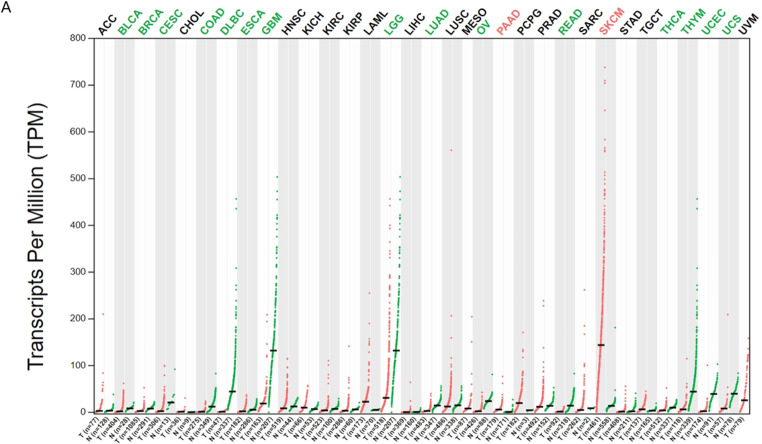
Differential expression of SNCA in Pan-cancers. According to this result, SNCA’s expression in a lot of cancers has changed significantly, and most of them are significantly down-regulated (**A**) (Green represents significant downregulation, while red represents significant upregulation). The types of cancer acronyms are as follows: adrenocortical carcinoma (ACC); acute myeloid leukemia (LAML); bladder urothelial carcinoma (BLCA); breast invasive carcinoma (BRCA); cervical squamous cell carcinoma (CESC); cholangiocarcinoma (CHOL); colon adenocarcinoma (COAD); lymphoid neoplasm diffuse large B cell lymphoma (DLBC); esophageal carcinoma (ESCA); glioblastoma multiforme (GBM); head and neck squamous cell carcinoma (HNSC); kidney chromophobe (KICH); kidney renal clear cell carcinoma (KIRC); kidney renal papillary cell carcinoma (KIRP); brain lower grade glioma (LGG); liver hepatocellular carcinoma (LIHC); lung adenocarcinoma (LUAD); lung squamous cell carcinoma (LUSC); mesothelioma (MESO); ovarian serous cystadenocarcinoma (OV); pancreatic adenocarcinoma (PAAD); pheochromocytoma and paraganglioma (PCPG); prostate adenocarcinoma (PRAD); rectum adenocarcinoma (READ); sarcoma (SARC); skin cutaneous melanoma (SKCM); stomach adenocarcinoma (STAD); testicular germ cell tumors (TGCT); thyroid carcinoma (THCA); thymoma (THYM); uterine corpus endometrial carcinoma (UCEC); uterine carcinosarcoma (UCS); uveal melanoma (UVM)

**Fig. 4:**
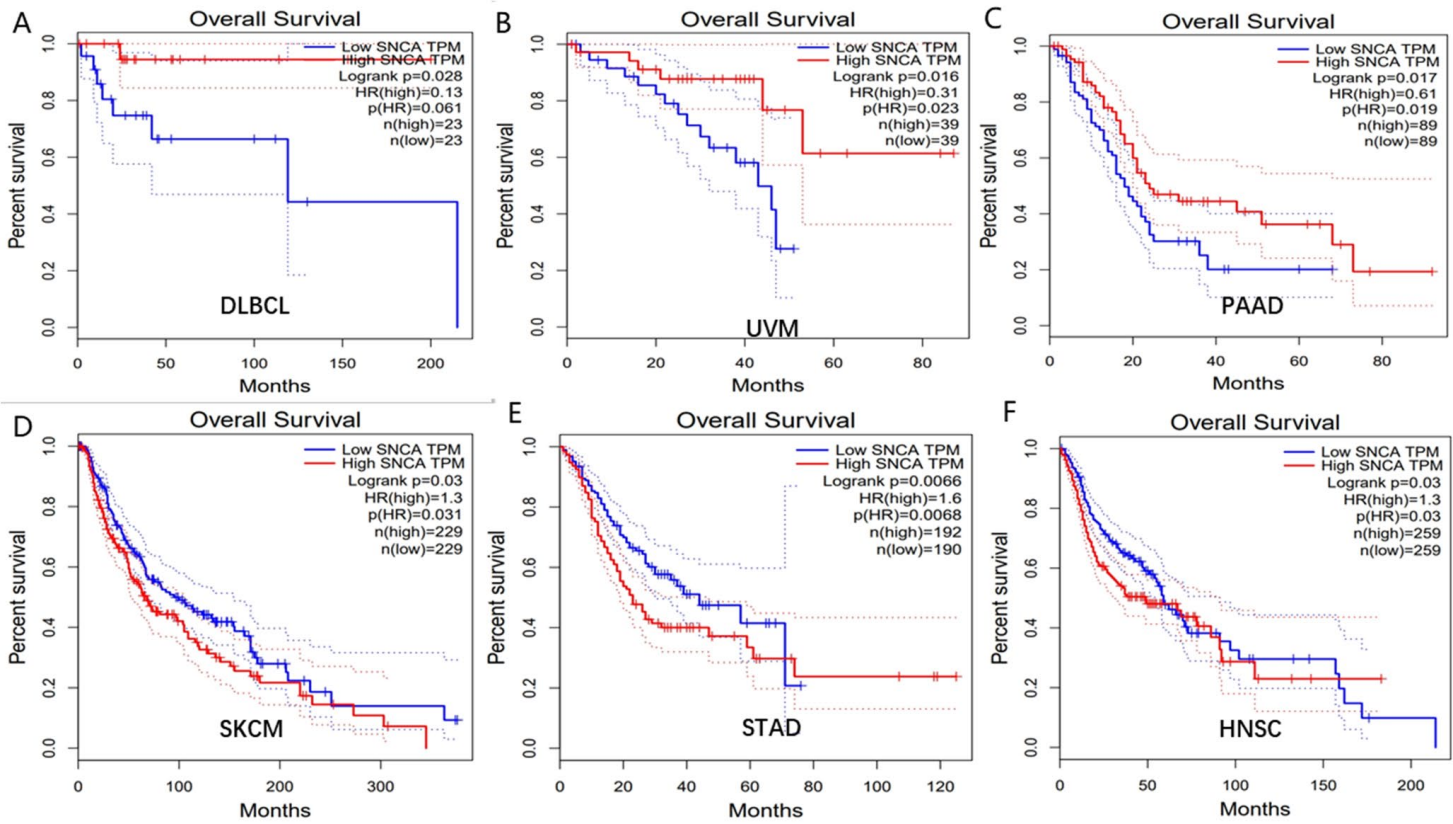
Prognostic value of SNCA in Pan-cancers. GEPIA online databases were used to analyze the prognostic value of SNCA in Pan-cancers. The Kaplan Meier survival curve showed that low expression of SNCA was significantly correlated with poor prognosis of DLBCL, UVM and PAAD (**A**-**C**), while high expression of SNCA was significantly correlated with poor prognosis of SKCM, STAD and HNSC (**D**-**F**)

GEPIA online databases were used to analyze SNCA’s expression level in different types of normal tissues and human cancer. we used GEPIA2 database, which integrated gene expression data from GTEx and TCGA to determine SNCA’s expression level in 337 normal tissues and 34 human tumors (Fig. 3A). According to this result, SNCA’s expression in a lot of cancers has changed significantly, and most of them are significantly down-regulated (Green represents significant downregulation, while red represents significant upregulation). The Kaplan Meier survival curve showed that low expression of SNCA was significantly correlated with poor prognosis of DLBCL, uveal melanoma (UVM) and pancreatic adenocarcinoma (PAAD) (Fig. 4A-C), while high expression of SNCA was significantly correlated with poor prognosis of skin cutaneous melanoma (SKCM), stomach adenocarcinoma (STAD) and head and neck squamous cell carcinoma (HNSC) (Fig. 4D-F). SNCA may therefore be a potential oncogene. Meanwhile, SNCA is significantly downregulated in COVID-19 patients, Our research results indicate that compared to the asymptomatic group, the SNCA expression in the symptomatic group is significantly downregulated (Figure S3). SNCA may be a potential therapeutic target for COVID-19 infection in cancer patients

### SNCA is a potential biomarker for DLBCL tissue and DLBCL sample

**Fig. 5:**
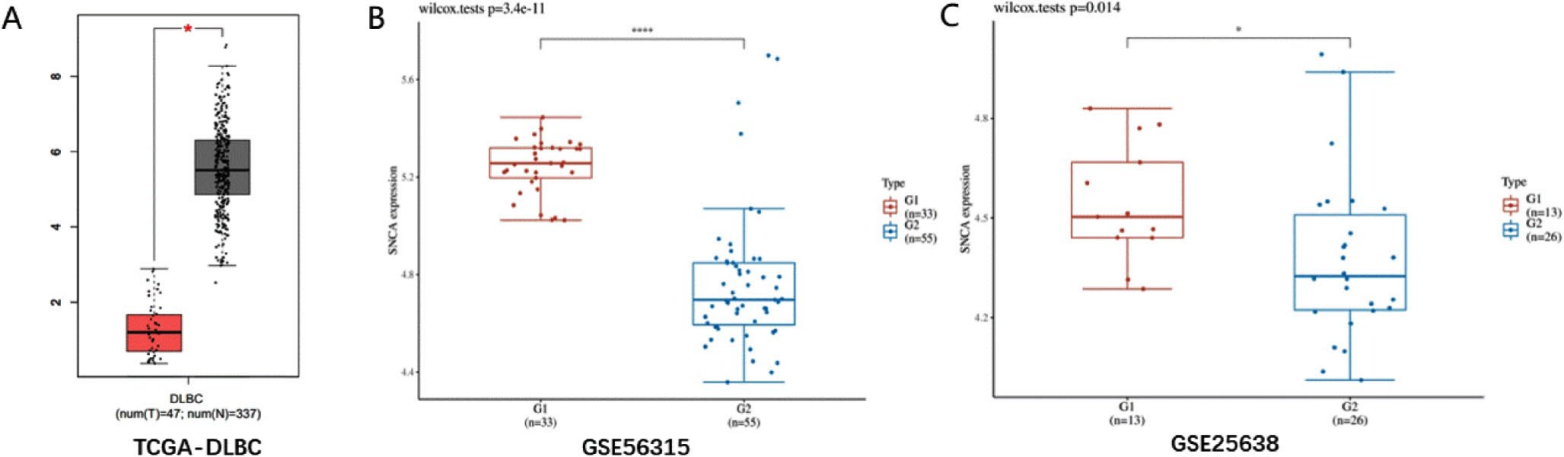
SNCA is a potential biomarker for DLBCL tissue and DLBCL sample. SNCA was significantly downregulated in TCGA-DLBC dataset (**A**), Red represents DLBCL samples, while black represents healthy samples. SNCA was significantly downregulated in GSE56315 dataset and GSE25638 dataset (**B**-**C**). G1 group and red represent healthy samples, G2 group and blue represent DLBCL samples, *P* < 0.05 is considered to have significant differences

Based on pan-cancer analysis and TCGA-DLBC dataset, we found that the expression level of SNCA was significantly downregulated in DLBCL samples (Figs. 3A and 5A). Based on two independent datasets (GSE56315 and GSE25638), we validated that the expression level of SNCA was significantly downregulated in DLBCL samples (Fig. 5B-C). Meanwhile, qPCR results of clinical samples showed that the expression level of SNCA was significantly downregulated in DLBCL samples compared to the healthy group (Figure S4).

### SNCA is a potential prognostic biomarker for DLBCL

In order to evaluate SNCA’s prognostic role in DLBCL, based on the TCGA-DLBC dataset, the prognosis of DLBCL patients after treatment was evaluated by analyzing SNCA’s high and low expression with Kaplan Meier survival curve, SNCA’s high expression therefore leads to a good prognosis in DLBCL patients (*P* < 0.05) (Fig. 4A). The results were validated on the GSE181063 dataset (Figure S5). The low expression level of SNCA promotes poor prognosis in patients, the high expression level of SNCA promotes good prognosis in patients.

### Construction of SNCA overexpression OCI-LY1 cell model

**Fig. 6:**
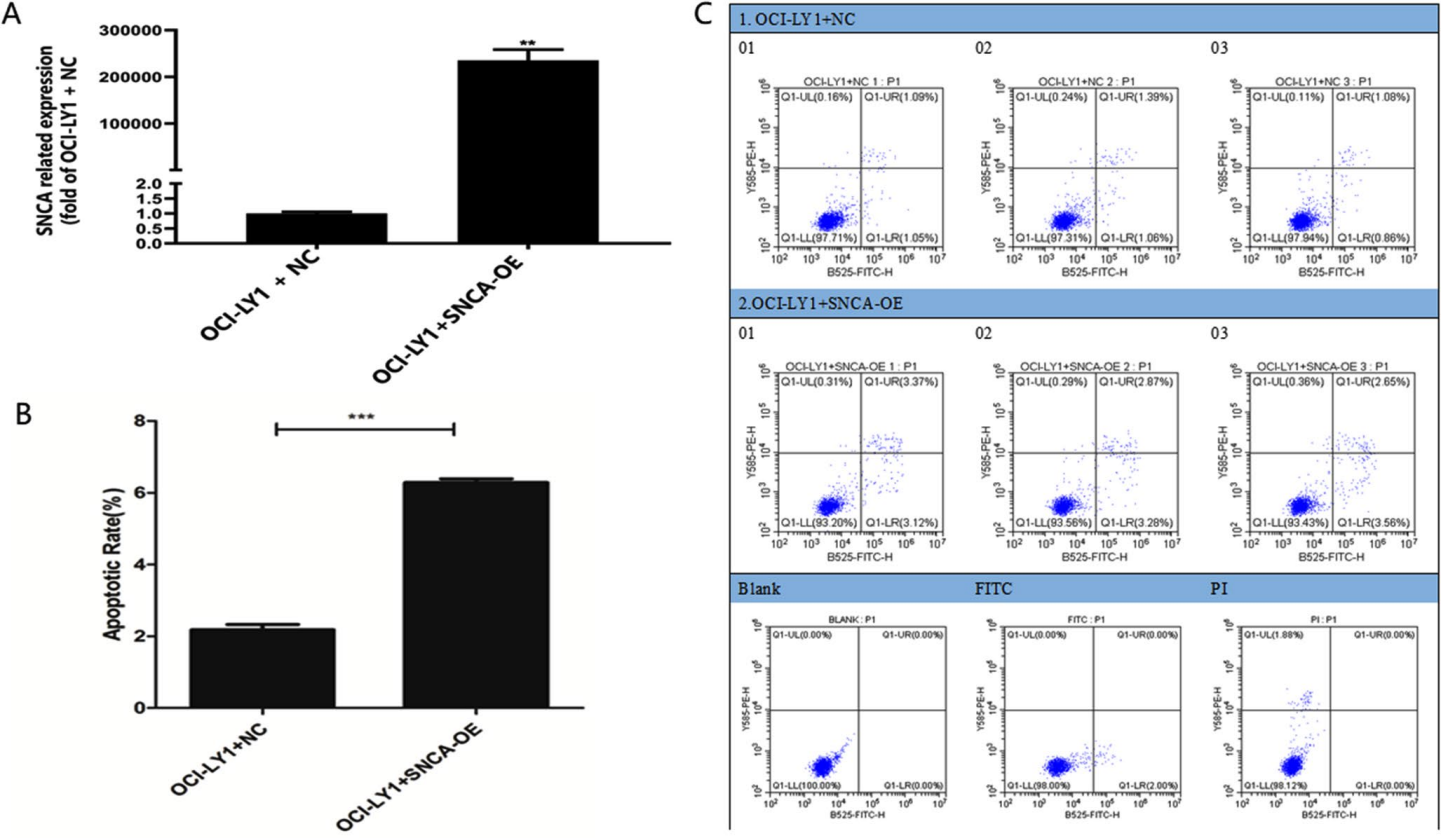
Construction of SNCA overexpression OCI-LY1 cell model and apoptosis experiments of OCI-LY1 cells. The qPCR results showed that compared with the control group, the cells transfected with OCI-LY1 + SNCA-OE had the significant overexpression effect on SNCA and named OCI-LY1 + SNCA-OE (**A**). The results of cell apoptosis showed that compared with the OCI-LY1 + NC group cells, the apoptosis rate of the OCI-LY1 + SNCA-OE group cells was significantly increased (**B**-**C**)

The qPCR results showed that compared with the control group, the cells transfected with OCI-LY1 + SNCA-OE had the significant overexpression effect on SNCA and named OCI-LY1 + SNCA-OE (Fig. 6A).

### Apoptosis experiments of OCI-LY1 cells

The results of cell apoptosis showed that compared with the OCI-LY1 + NC group cells, the apoptosis rate of the OCI-LY1 + SNCA-OE group cells was significantly increased (Fig. 6B-C). Therefore, the high expression of SNCA significantly promoted apoptosis of OCI-LY1 cells.

### Cycle experiments of OCI-LY1 cells

**Fig. 7:**
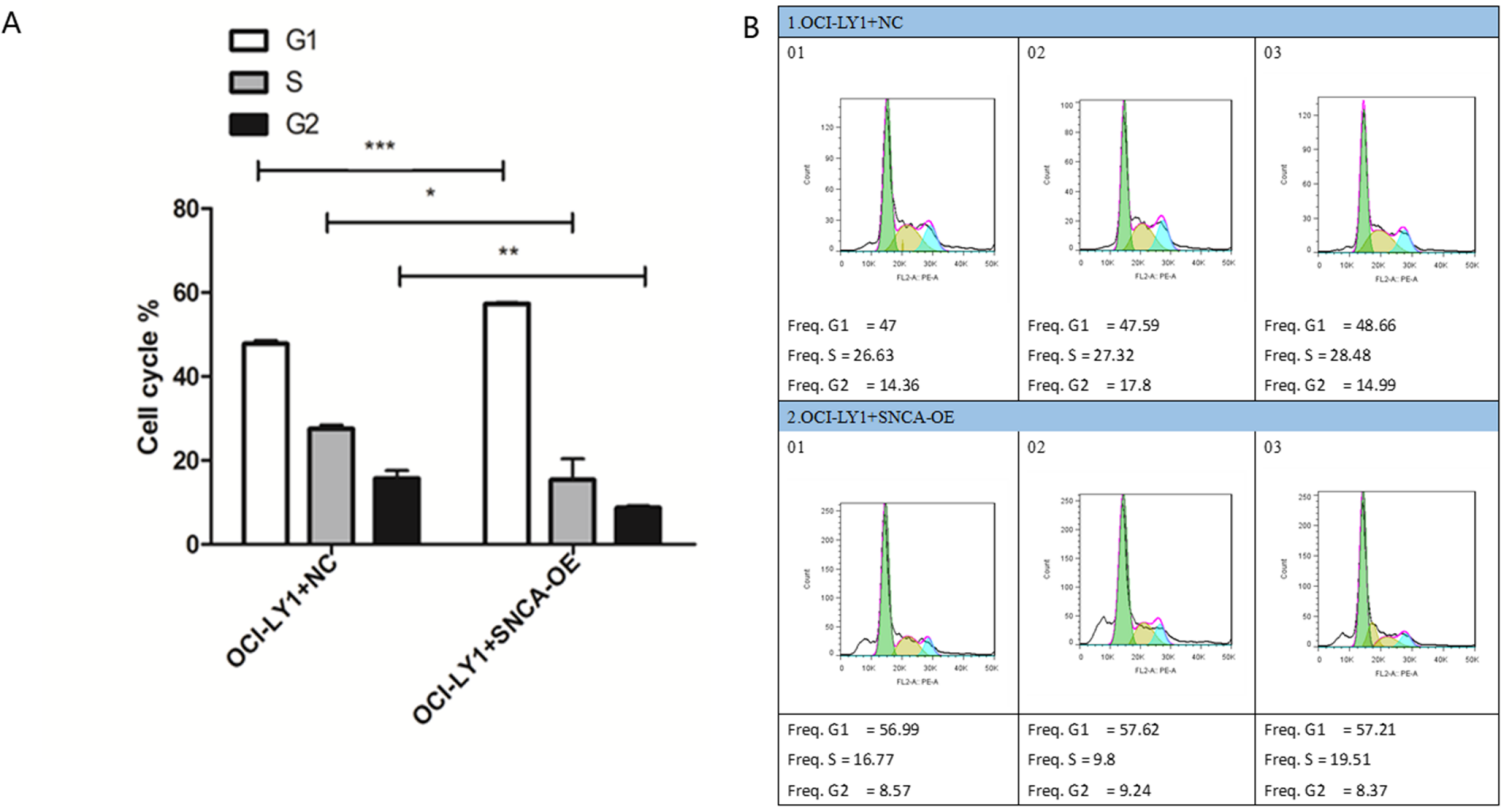
Cycle experiments of OCI-LY1 cells. The cell cycle results showed that compared with the OCI-LY1 + NC group, the OCI-LY1 + SNCA-OE group showed a significant increase in G1 phase cells, a significant decrease in S phase cells, and a significant decrease in G2 phase cells (**A**-**B**)

The cell cycle results showed that compared with the OCI-LY1 + NC group, the OCI-LY1 + SNCA-OE group showed a significant increase in G1 phase cells, a significant decrease in S phase cells, and a significant decrease in G2 phase cells (Fig. 7A-B). Therefore, the high expression of SNCA significantly inhibited the progression of OCI-LY1 cell cycle.

### Verification of differential expression levels of SNCA in COVID-19 infected DLBCL patients

The qPCR results showed that compared with the control group, the expression level of SNCA was significantly downregulated in COVID-19 infected DLBCL patients group (Figure S6).

### Pathway analysis of SNCA at the transcriptional level in COVID-19 and DLBCL

To analyze the common pathway of the SNCA in DLBCL and COVID-19, we screened genes closely related to SNCA based on the GSE177477 dataset and TCGA-DLBC dataset (correlation coefficient | R |>0.3 and *P* < 0.05). In the GSE177477 dataset, a total of 3578 positively associated genes and 6804 negatively associated genes were selected. The GSEA analysis results show that the high expression of SNCA can activate Regulation of immune system process and T cell receptor signaling pathway; high expression of SNCA can inhibit Mitotic cell cycle, Regulation of cyclin dependent protein kinase activity and Response to oxidative stress (Figure S7A). In the TCGA-DLBC dataset, a total of 3913 positively associated genes and 239 negatively associated genes were selected. The GSEA analysis results show that the high expression of SNCA can activate Regulation of immune system process, T cell receptor signaling pathway and Response to oxidative stress; high expression of SNCA can inhibit Mitotic cell cycle and Regulation of cyclin dependent protein kinase activity (Figure S7B).

### Correlation analysis between SNCA and immune cell infiltration in COVID-19 and DLBCL

**Fig. 8:**
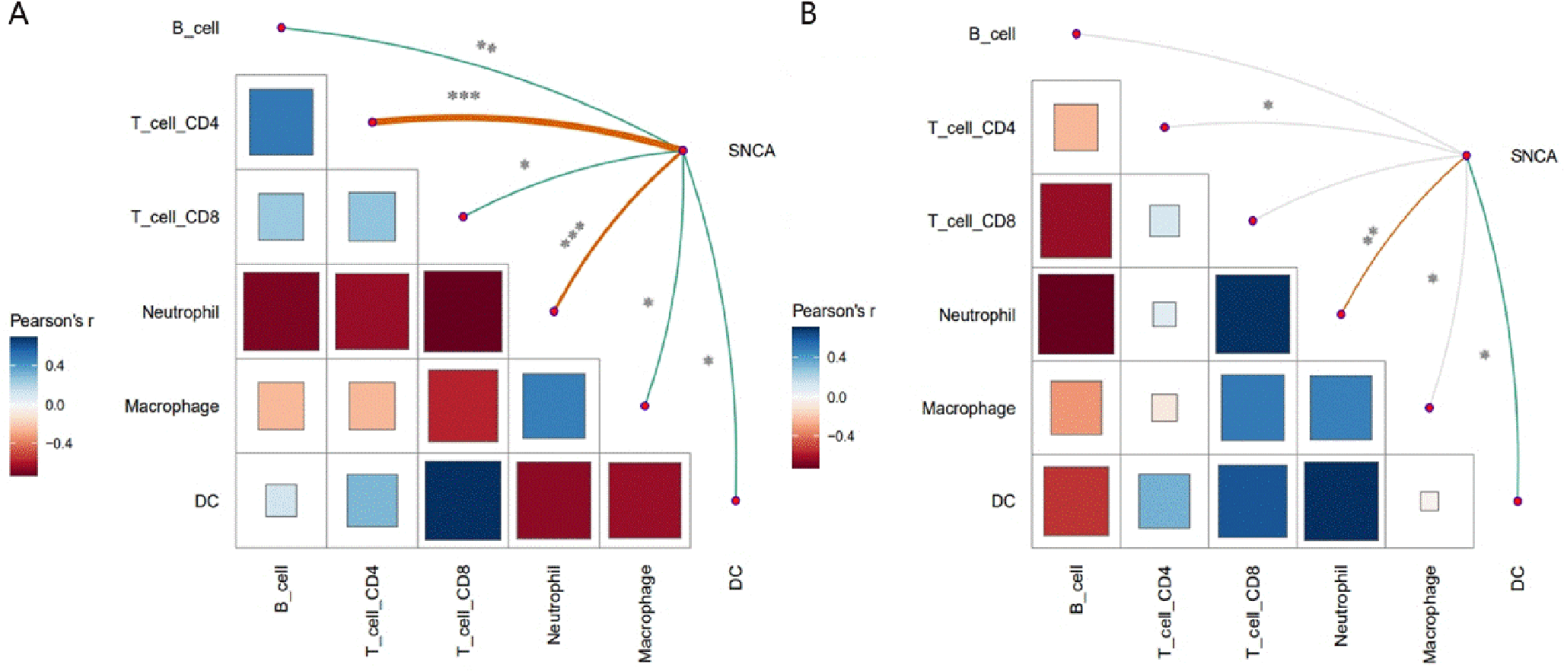
Correlation analysis between SNCA and immune cell infiltration in COVID-19 and DLBCL. In the GSE177477 dataset, we found that SNCA is significantly correlated with infiltration of six types of immune cells (CD4 + T cells, B cells, CD8 + T cells, neutrophils, dendritic cells and macrophages), SNCA is significantly positively related to dendritic cells infiltration, CD4 + T cells, B cells and CD8 + T cells; SNCA is significantly negatively related to neutrophils and macrophages (**A**). In the TCGA-DLBC dataset, we found that SNCA is significantly positively related to dendritic cells infiltration, neutrophils and macrophages; SNCA is significantly negatively related to CD4 + T cells (**B**). *P < 0.1, **P < 0.01, ***P < 0.001

We used TIMER to determine whether the expression of SNCA in COVID-19 and DLBCL is related to immune cell infiltration. In the GSE177477 dataset, we found that SNCA is significantly correlated with infiltration of six types of immune cells (CD4 + T cells, B cells, CD8 + T cells, neutrophils, dendritic cells and macrophages), SNCA is significantly positively related to DCs, CD4 + T cells, B cells and CD8 + T cells; SNCA is significantly negatively related to neutrophils and macrophages (Fig. 8A). In the TCGA-DLBC dataset, we found that SNCA is significantly positively related to DCs, neutrophils and macrophages; SNCA is significantly negatively related to CD4 + T cells (Fig. 8B). *P < 0.1, **P < 0.01, ***P < 0.001.

## Discussion

COVID-19 is an infectious disease caused by SARS-CoV-2, which has serious impacts on human life, health, and socio-economic development [1]. At present, the mechanism of COVID-19 has not been fully elucidated [33]. The study found that the serum copper ion level of COVID-19 patients increased [15], and compared with non-severe patients, the whole blood copper ion level of severe COVID-19 patients significantly increased [16]. Therefore, copper homeostasis in COVID-19 is unbalanced. The imbalance of copper ion levels can disrupt certain mitochondrial metabolic enzymes, which can induce cell death, namely cuprotosis, on the other hand, the key signaling pathway of cuprotosis, FDX1 (Ferredoxin 1) - LIAS (Lipic Acid Synthetase) axis plays an important regulatory role in cellular oxidative stress, which can lead to cell apoptosis and the production of reactive oxygen species (ROS) [14]. It is reported that the ROS in patients with COVID-19 increases [34], so the level of copper ions in patients with COVID-19 is unbalanced, thus inducing cuprotosis, and further regulating oxidative stress through the FDX1-LAS axis, leading to the increase of ROS in the body. According to reports, CRGs play an important role in COVID-19 [19]. Through univariate analysis, machine learning, and clinical sample detection, we found that SNCA is a key differentially expressed in COVID-19 patients. Our study found that the expression of SNCA in peripheral blood samples of patients with severe COVID-19 was significantly lower than that of asymptomatic patients. The SNCA gene expresses a protein rich in neurons- α- Synuclein [35], defined as copper binding protein, is involved in the copper death pathway [36]. Our pathway analysis found that SNCA was involved in the oxidative stress pathway in patients with COVID-19. Therefore, SNCA may regulate the copper death pathway of patients with COVID-19, and further regulate oxidative stress through the FDX1-LAS axis, leading to the change of ROS in patients with COVID-19.

There are many complex interactions between COVID-19 and cancer [8]. The level of copper ions in the body of patients with COVID-19 is unbalanced. The imbalance of copper homeostasis in the body is closely related to the occurrence and development of cancer. The level of copper ions in different types of tumor tissue samples shows an upward trend, including DLBCL, lung cancer and digestive tract kidney cancer [17]. The level of copper ions in patients with COVID-19 and cancer increases, and excessive copper ions can induce cuprotosis [18], so cuprotosis plays a complex role in the development of COVID-19 and cancer. CRGs have been proved to be potential therapeutic targets for COVID-19 and cancer [19, 20]. COVID-19 and DLBCL share a common signal crosstalk mechanism [13] and cuprotosis related DLD gene have been proved to be potential therapeutic targets for DLBCL patients infected with COVID-19 [21]. SNCA has been reported to be related to the occurrence and development of various cancers. In breast cancer tissues, the expression level of SNCA is low, in vitro and in vivo experiments, the overexpression of SNCA blocks the metastasis of breast cancer [36]. In bladder cancer tissues, SNCA is significantly down regulated, and is significantly related to DNA methylation and T cell immune infiltration of bladder cancer, which is an independent prognostic factor of bladder cancer [37]. The expression level of SNCA in LUAD tissue is lower than that in normal tissue, and high expression of SNCA is associated with better prognosis. SNCA can inhibit the growth of A549 cells by inhibiting the activity of the PI3K/AKT/mTOR pathway [38]. SNCA is associated with the prognosis and immune invasion of pancreatic cancer [39]. Our pan-cancer analysis found that SNCA expression is downregulated in various cancers and significantly correlated with the prognosis of various cancers. We validated the differential expression and prognostic correlation of SNCA through clinical samples and multiple independent datasets. DLBCL cells confirmed that high expression of SNCA can significantly inhibit cycle progression and promote cell apoptosis. Therefore, SNCA is a potential therapeutic target for DLBCL.

We verified the differential expression of SNCA in DLBCL patients infected with COVID-19 by qPCR detection of clinical samples. The GSEA analysis results show that the high expression of SNCA can activate Regulation of immune system process and T cell receptor signaling pathway in COVID-19 and DLBCL; high expression of SNCA can inhibit Mitotic cell cycle and Regulation of cyclin dependent protein kinase activity in COVID-19 and DLBCL, therefore, SNCA may participate in the crosstalk mechanism of COVID-19 and DLBCL through cell cycle and immune pathways. At the cellular functional level, high expression of SNCA can inhibit the cell cycle crosstalk pathway of COVID-19 and DLBCL, thereby inhibiting the cycle progression of DLBCL. Our cell experiments also confirm that high expression of SNCA significantly inhibits the cycle progression of DLBCL. Immune infiltration analysis showed that SNCA was significantly positively correlated with the immune infiltration level of DCs in COVID-19 and DLBCL. DCs were mainly responsible for antigen presentation, T cell activation, and immune regulation [40]. Due to the unique ability of DCs to stimulate initial T cells, targeted therapy for DCs has always been a hot topic in the field of cancer treatment. The most crucial role of DCs is to regulate the protein antigen presentation mediated by the major histocompatibility complex (MHC) to activate specific T cells [41]. We found that in DLBCL and COVID-19, highly expressed SNCA can activate T cell receptor (TCR) signaling pathway. Due to the fact that TCR only binds to MHC-antigen peptide complexes [42], therefore, in the crosstalk mechanism between COVID-19 and DLBCL, highly expressed SNCA may regulate MHC mediated protein antigen presentation through DCs, thereby activating the TCR signaling pathway, enhancing T cell-mediated anti-tumor immunity, and clearing DLBCL cells.

There inevitably are several limitations to this study. Firstly, we are unable to obtain relevant clinical data on COVID-19 infected DLBC patients from the database. We collected clinical samples and qPCR results confirmed that the expression levels of SNCA were significantly downregulated in DLBCL patients, COVID-19 patients and COVID-19 infected DLBCL patients. Due to the limited number of clinical samples collected, we were unable to analyze the clinical correlation between SNCA and COVID-19 infected DLBCL patients. In the next study, we will include single center and multicenter clinical samples and information to further investigate the clinical diagnostic and therapeutic value of SNCA in COVID-19 infected DLBCL patients. Secondly, although SNCA’s function has been confirmed by DLBCL cells, its downstream and upstream mechanisms are not yet clear, and further experiments are needed to study its mechanism, which will be the research we need in the future.

## Conclusion

Firstly, we screened the key cuproptosis-related SNCA gene through univariate analysis, machine learning, two independent COVID-19 dataset and clinical samples. Secondly, we determined the differential expression and prognostic relevance of SNCA in DLBCL patients through pan-cancer analysis. We validated the differential expression and prognostic relevance of SNCA through clinical samples and multiple independent datasets and validated the functional mechanism of SNCA in DLBCL through cell experiments. Therefore, SNCA is a potential therapeutic target for DLBCL. Finally, we validated the differential expression of SNCA in COVID-19 infected DLBCL patients through qPCR detection of clinical samples and elucidated the common mechanism of SNCA in COVID-19 and DLBCL.

## Electronic supplementary material

Below is the link to the electronic supplementary material.

Supplementary Material 1

## Data Availability

No datasets were generated or analysed during the current study.
