# Supplementary material for: SNCA is a potential therapeutic target for COVID-19 infection in diffuse large B-cell lymphoma patients
Source: Apoptosis. 2024 Jul 15;29(9-10):1454–65. doi: 10.1007/s10495-024-01996-9 (PMC11416394; doi:10.1007/s10495-024-01996-9)
Supplement: Supplementary file 1 — Supplementary Material 1 [file 10495_2024_1996_MOESM1_ESM.docx]

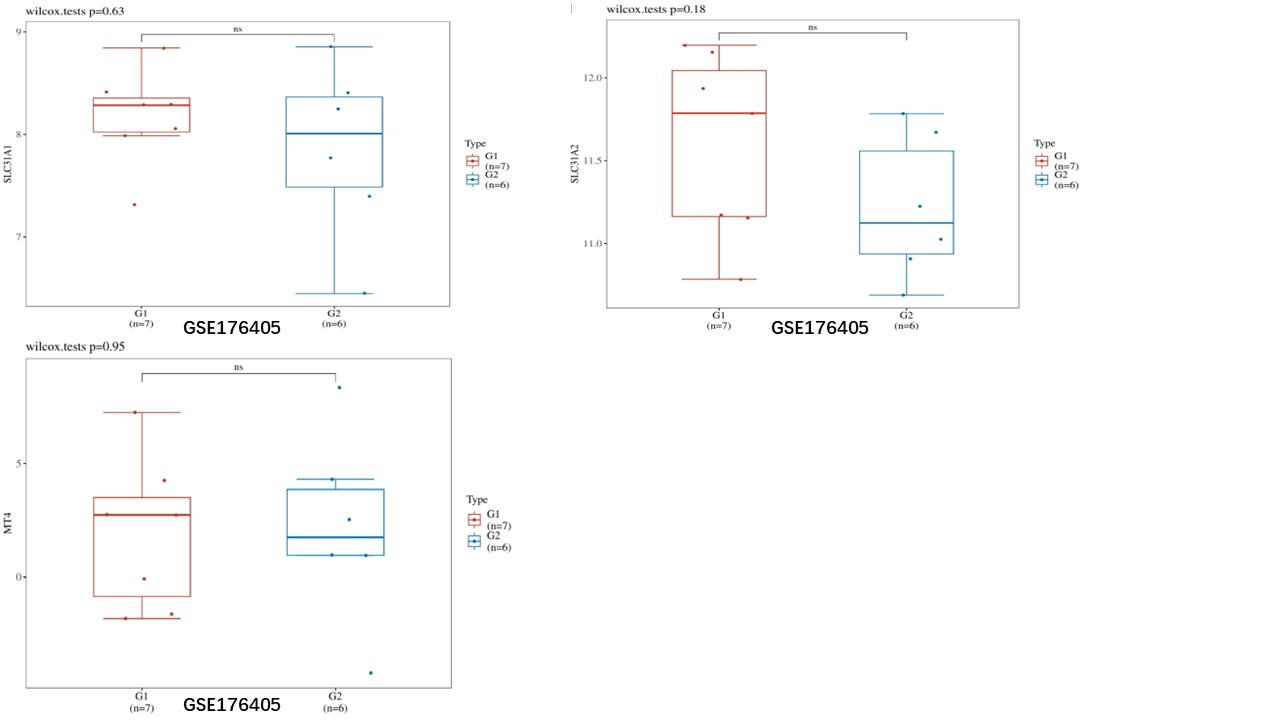


**Figure S1 Independent dataset to validate key genes of COVID-19**

SLC31A1, SLC31A2 and MT4 are not significantly differentially expressed in the GSE176405 dataset (G1 group and red represent healthy samples, G2 group and blue represent COVID-19 samples, P<0.05 is considered to have no significant differences).


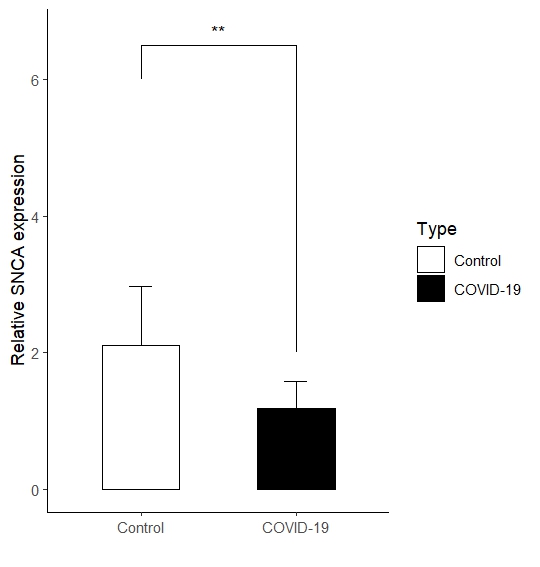


**Figure S2 qPCR detection of SNCA expression levels in the control group and COVID-19 patients group.**

The qPCR results showed that SNCA was significantly downregulated in COVID-19 patients compared to the control group. White represents the control group, black represents the COVID-19 patient group, P<0.05 and FC<0.67 are considered to have significant differences.


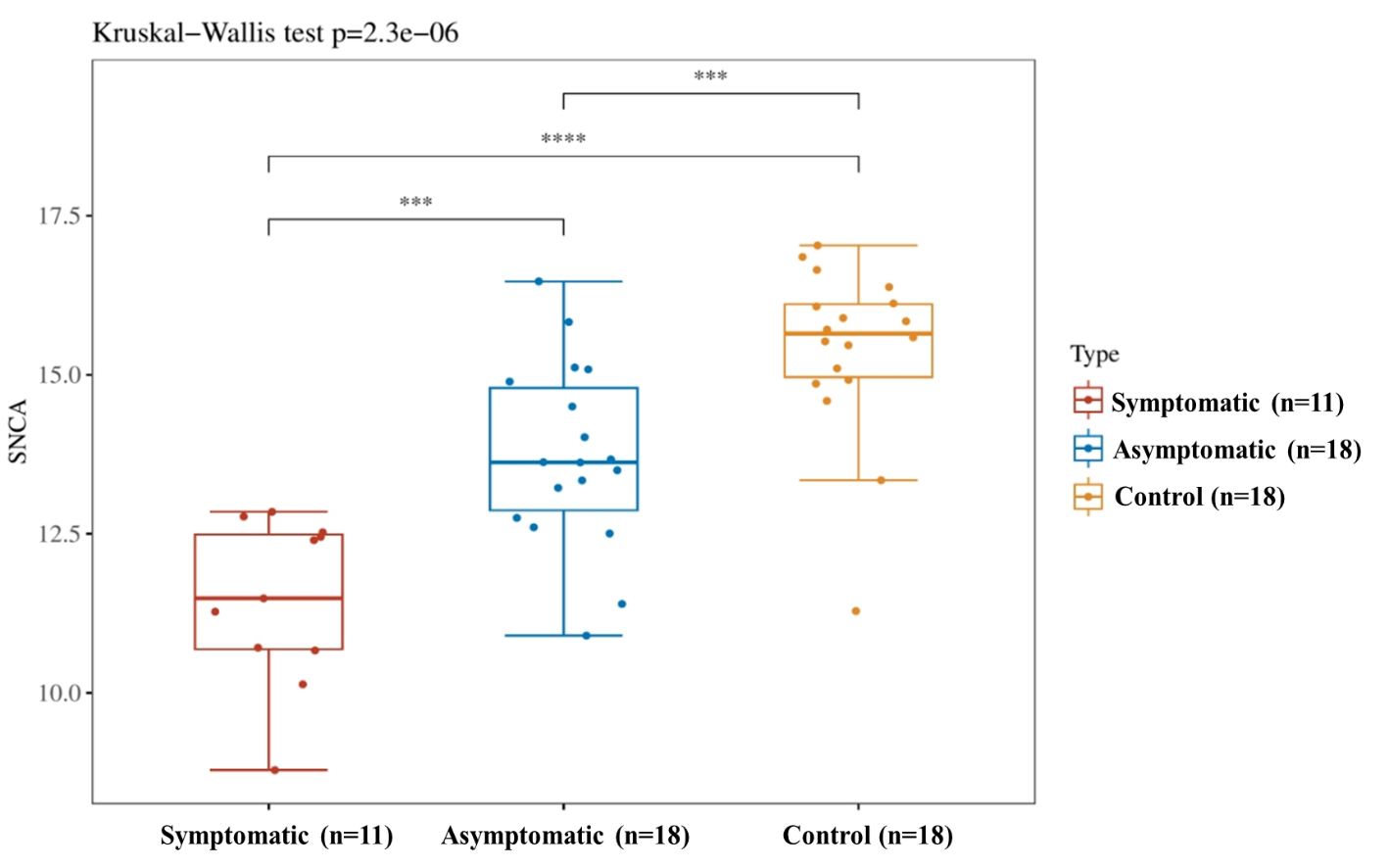


**Figure S3 Differential analysis of SNCA expression levels among the control group, asymptomatic group and symptomatic group.**

Compared with the control group, the expression level of SNCA was significantly downregulated in both asymptomatic and symptomatic patients; Compared to asymptomatic patients, the expression level of SNCA is significantly downregulated in symptomatic patients. P<0.05 are considered to have significant differences.


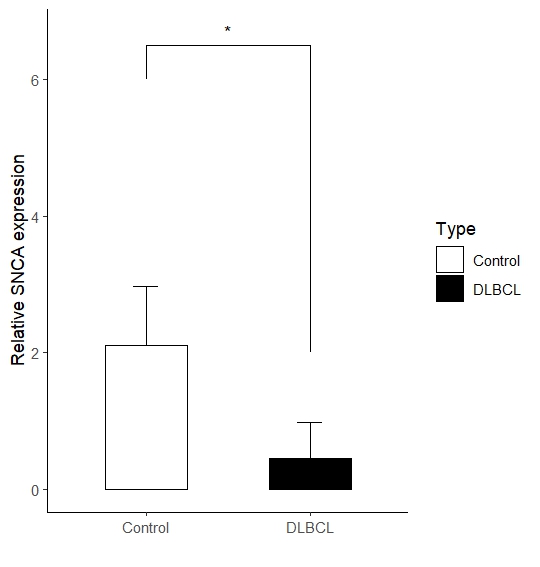


**Figure S4 qPCR detection of SNCA expression levels in the control group and DLBCL patients group.**

The qPCR results showed that SNCA was significantly downregulated in DLBCL patients compared to the control group. White represents the control group, black represents the DLBCL patient group, P<0.05 and FC<0.67 are considered to have significant differences.


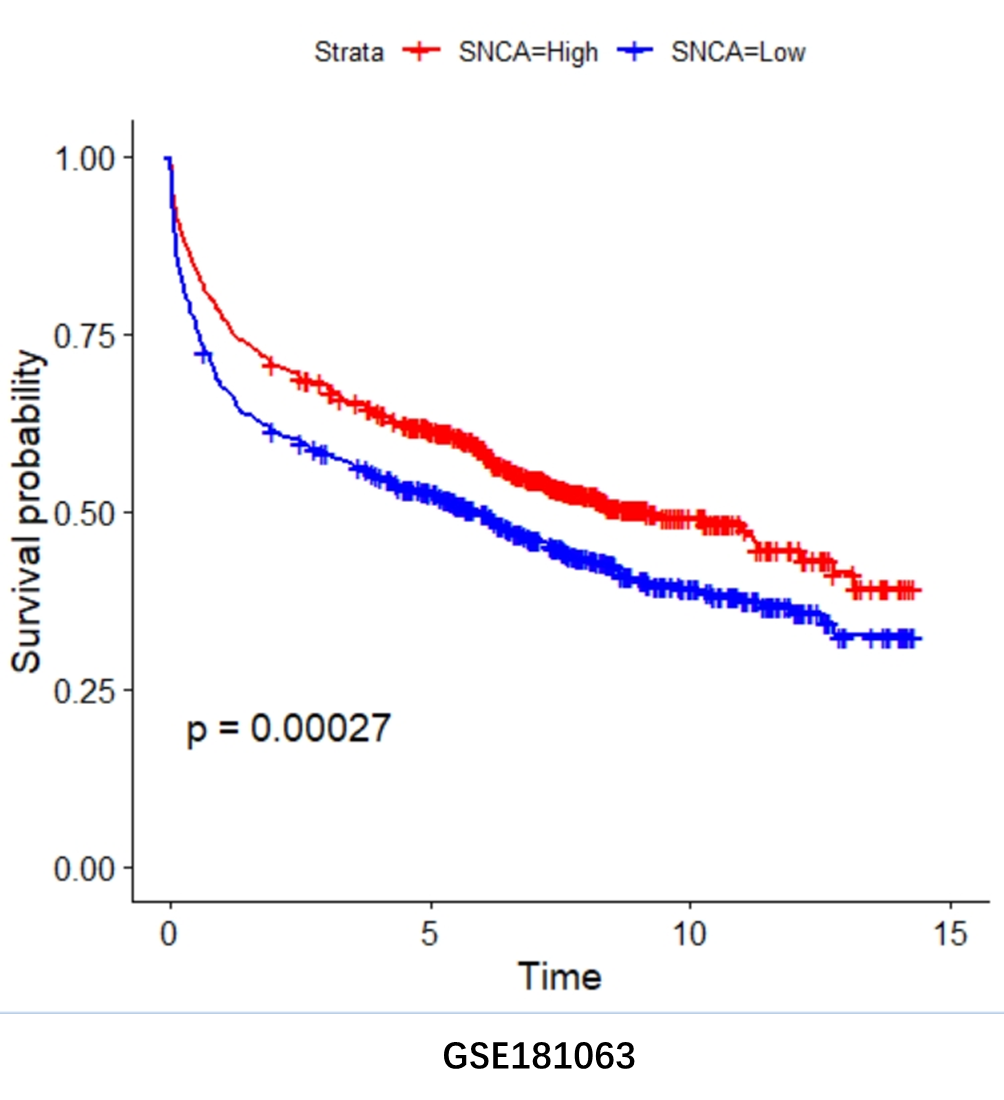


**Figure S5 Based on GSE181063 data, we evaluated the prognostic value of SNCA in DLBCL.**

Based on GSE181063 data, we evaluated the prognostic value of SNCA in DLBCL and found that low expression of SNCA was significantly associated with poor prognosis in DLBCL patients. Red represents the high expression group, blue represents the low expression group, P<0.05 are considered to have significant differences.


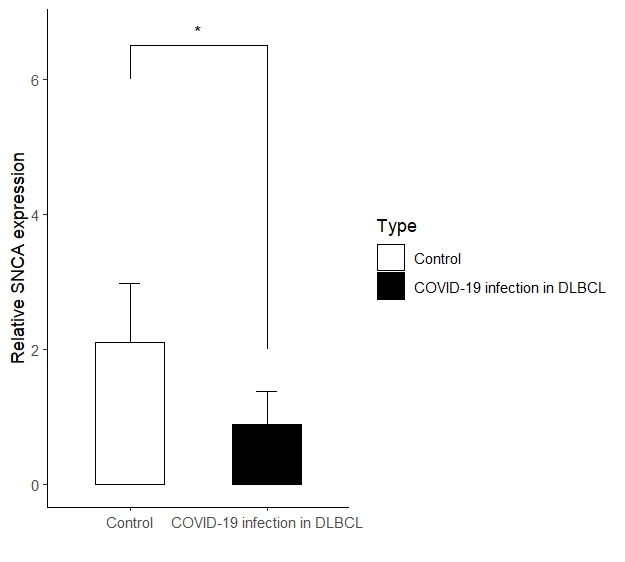


**Figure S6 qPCR detection of SNCA expression levels in the control group and** **COVID-19 infection in DLBCL patients group.**

The qPCR results showed that SNCA was significantly downregulated in COVID-19 infection in DLBCL patients group compared to the control group. White represents the control group, black represents the COVID-19 infection in DLBCL patients group, P<0.05 and FC<0.67 are considered to have significant differences.


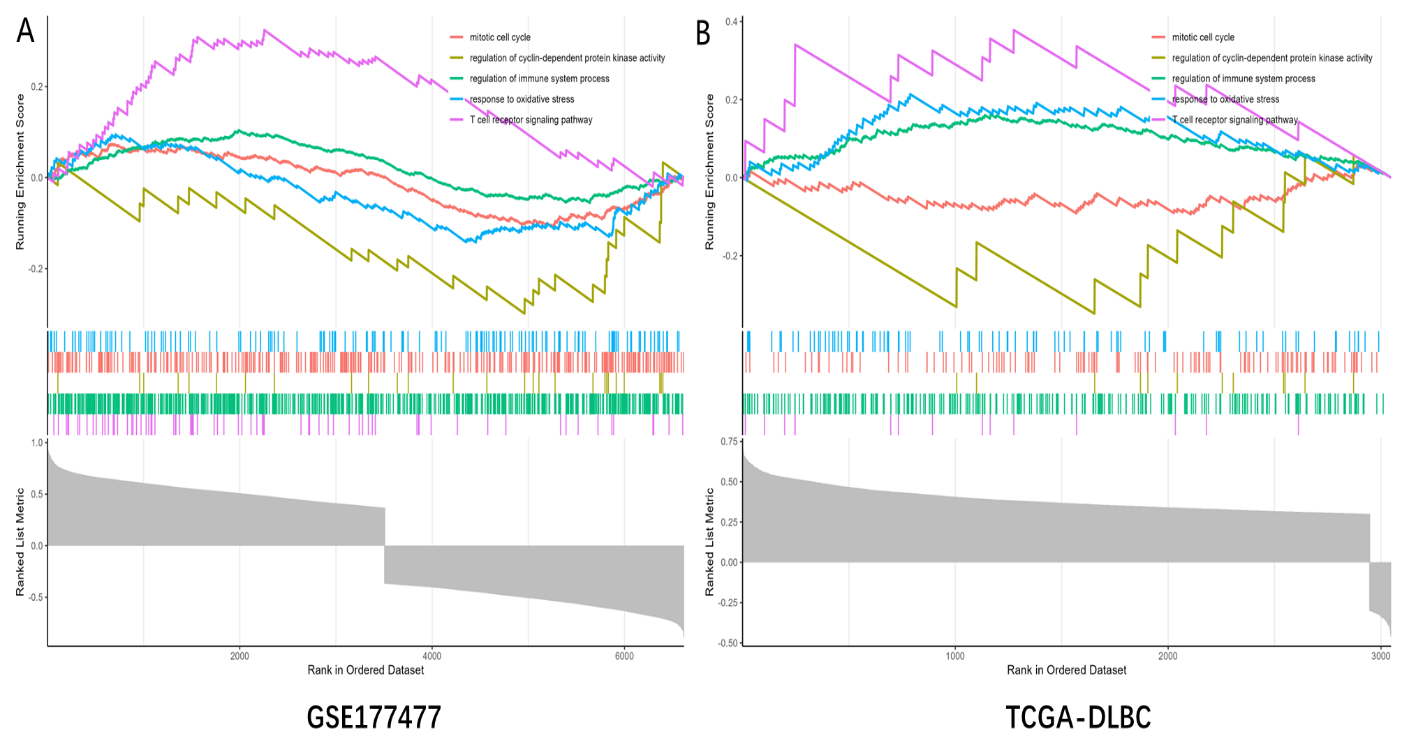


**Figure S7 Pathway analysis of SNCA at the transcriptional level in COVID-19 and DLBCL.**

In the GSE177477 dataset, a total of 3578 positively associated genes and 6804 negatively associated genes were selected. The GSEA analysis results show that the high expression of SNCA can activate Regulation of immune system process and T cell receptor signaling pathway; high expression of SNCA can inhibit Mitotic cell cycle, Regulation of cyclin dependent protein kinase activity and Response to oxidative stress (A). In the TCGA-DLBC dataset, a total of 3913 positively associated genes and 239 negatively associated genes were selected. The GSEA analysis results show that the high expression of SNCA can activate Regulation of immune system process, T cell receptor signaling pathway and Response to oxidative stress; high expression of SNCA can inhibit Mitotic cell cycle and Regulation of cyclin dependent protein kinase activity (B).
